# Supplementary material for: Adolescent self-consent for vaccinations: protocol for a mixed methods systematic review
Source: BMJ Open. 2018 May 20;8(5):e021335. doi: 10.1136/bmjopen-2017-021335 (PMC5961593; doi:10.1136/bmjopen-2017-021335)
Supplement: Author's manuscript [file bmjopen-2017-021335.draft_revisions.pdf]

BMJ Open

BMJ Open is committed to open peer review. As part of this commitment we make the peer review history of every article we publish publicly available.

When an article is published we post the peer reviewers' comments and the authors' responses online. We also post the versions of the paper that were used during peer review. These are the versions that the peer review comments apply to.

The versions of the paper that follow are the versions that were submitted during the peer review process. They are not the versions of record or the final published versions. They should not be cited or distributed as the published version of this manuscript.

BMJ Open is an open access journal and the full, final, typeset and author-corrected version of record of the manuscript is available on our site with no access controls, subscription charges or pay-per-view fees (http://bmjopen.bmj.com).

If you have any questions on BMJ Open's open peer review process please email info.bmjopen@bmj.com

BMJ Open

Adolescent self-consent for vaccinations: protocol for a mixed methods systematic review

Journal:	*BMJ Open*
Manuscript ID	bmjopen-2017-021335
Article Type:	Protocol
Date Submitted by the Author:	29-Dec-2017
Complete List of Authors:	Batista, Harriet; University of Bristol, Population Health Sciences: Bristol Medical School<br>Hickman, M; University of Bristol, Population Health Sciences: Bristol Medical School<br>Macleod, John; University of Bristol, Population Health Sciences: Bristol Medical School<br>Audrey, Suzanne; University of Bristol, Population Health Sciences: Bristol Medical School
Keywords:	Self-consent, Vaccination, Systematic review, Mixed methods, Adolescents

SCHOLARONE™
Manuscripts

For peer review only

TITLE: Adolescent self-consent for vaccinations: protocol for a mixed methods systematic review

Dr Harriet Batista Ferrer, Population Health Sciences, Bristol Medical School, University of Bristol.

Professor Matthew Hickman, Population Health Sciences, Bristol Medical School, University of Bristol.

Professor John Macleod, Population Health Sciences, Bristol Medical School, University of Bristol.

Dr Suzanne Audrey, Population Health Sciences, Bristol Medical School, University of Bristol.

Corresponding author:

Dr Harriet Batista Ferrer

Senior Research Associate

Population Health Sciences, Bristol Medical School

University of Bristol

Canynge Hall

Whatley Road

Bristol BS8 2PS

Email: Harriet.Batista@bristol.ac.uk

Telephone: +44 (0) 117 928 7223

Word count: 2,219

Key words

Self-consent, Vaccination, Systematic review, Adolescents, Mixed Methods

For peer review only

ABSTRACT

Introduction: The recent global expansion of routine adolescent vaccination programmes has the potential to protect young people against the acquisition of infectious disease and improve their health. Although in many countries the legal framework supports young people to provide consent for medical interventions if they are considered competent, written parental consent can act as a barrier to uptake as it is frequently a condition of adolescent vaccination programmes. The aim of this systematic review protocol is to document the methods which will be used to identify, appraise and synthesise the available qualitative and quantitative evidence to address: (i) whether implementation of adolescent self-consent procedures can increase vaccination uptake, and; (ii) the barriers and facilitators to implementation of adolescent self-consent procedures.

Methods and analysis: Comprehensive search strategy of all relevant electronic databases for both qualitative and quantitative studies using predefined inclusion and exclusion criteria. At least two authors will independently review titles and abstracts, extract data and assess the methodological quality of eligible primary studies, resolving disagreements by consensus. Quantitative studies will be reported narratively and where possible pooled in a meta-analysis using a random-effects model. The findings of qualitative primary studies will be extracted, interpreted and synthesised to identify overarching themes as well as similarities and differences within those themes.

Ethics and dissemination: As this systematic review involves analysis of secondary data, the study does not require ethical approvals. We will use our findings to assess whether the evidence supports the hypothesis that self-consent procedures can increase coverage of adolescent vaccination programmes. We will identify barriers and facilitators to the implementation of adolescent self-consent for vaccination, and make recommendations for policy-makers and practitioners in relation to consent procedures within vaccination programmes for young people.

Systematic review registration: PROSPERO CRD42017084509

Word count: 284

For peer review only

Strengths and limitations of this study

- The mixed methods systematic review will answer complementary research questions about self-consent for adolescent vaccination programmes
- Robust systematic review methodology will be used to identify, appraise and synthesise the relevant qualitative and quantitative literature
- Improvement to uptake of adolescent vaccination programmes by introduction of self-consent procedures will be assessed in quantitative studies
- Synthesis of qualitative studies will examine barriers and facilitators to self-consent for adolescent vaccination programmes
- Findings from this mixed-methods systematic review will inform recommendations for future policy and practice

For peer review only

INTRODUCTION

In recent years, the number of routine vaccinations recommended during adolescence have increased, and include vaccines that protect against tetanus, diphtheria, meningitis and human papillomavirus (HPV) acquisition [1, 2]. Provided sufficient coverage is achieved, the expansion of adolescent vaccination programmes may improve young people's health by protecting them from potentially life- threatening infectious diseases.

The introduction of new adolescent vaccination programmes is relevant to the debate about young people's capacity to provide consent to receive medical treatment. The United Nations Convention on the Rights of the Child recognises the right for all children and young people to participate in decision-making processes which involve them [3]. However, the World Health Organisation (WHO) has acknowledged difficulties over consent for vaccination of adolescents because of their age, and describes current practice through which countries are encouraged to adopt procedures that ensure parents have been informed and agreed to the vaccination [4].

In most countries, the legal framework for consent requires parental or guardian permission for young people aged below 18 years [4]. However, the age of consent for medical interventions, such as vaccination programmes, is lower in some countries. In the United Kingdom (UK), Canada and Sweden young women are legally able to override parental decisions if they are considered mature enough to make, and understand the consequences of, the decision to vaccinate. In Australia and the United States of America (USA) there are geographic variations of the age (12 to 17 years) that a young person can consent to be vaccinated. Despite young people being supported by the law to provide consent themselves, written parental consent is usually sought. In relation to the HPV vaccination programme, this has been shown to act as an important barrier preventing young women (usually aged 12 to 13 years) receiving the Human Papillomavirus (HPV) vaccine, with

For peer review only

implications for vaccination programme coverage [5, 6]. Furthermore, it is a barrier with potential to reinforce health inequalities since lack of written parental consent may also be related to lower socioeconomic status and some ethnic groups [5, 7].

To examine the issue of self-consent for the HPV vaccine in more detail, a mixed-methods study has been funded by the National Institute for Health Research (NIHR) Research for Patient Benefit Programme (RfPB) in England. The study is examining the practicality, acceptability and impact of implementing new self-consent procedures for the schools-based HPV vaccination in two local authorities in the south-west of England [8]. There are three elements to the study: statistical analyses of routine data to assess the impact of self-consent on overall uptake levels and in relation to socio-economic status, ethnicity and type of school; a process evaluation to examine the context, implementation and response to the new consent procedures, and; a systematic review of the evidence relating to self-consent for adolescent vaccines. The current protocol focusses on the systematic review which will run alongside, and inform, the other elements of the study.

An initial scoping search suggested a paucity of peer-reviewed evidence in relation to self-consent procedures for HPV vaccination programmes. Since issues relating to self-consent for the HPV vaccination are likely to be relevant for other vaccinations delivered during adolescence we widened the scope of the systematic review to identify and collate the evidence across all adolescent vaccination programmes. We chose to restrict to vaccination programmes, rather than include studies related to healthcare in general, to ensure the findings were relevant to the programme of research described above. Therefore, the aim of this mixed-methods systematic review is to identify, appraise and synthesise the available qualitative and quantitative literature to gain understanding as to: (i) whether implementation of adolescent self-consent procedures can increase vaccination uptake, and; (ii) the related barriers and facilitators to implementation of adolescent self-consent procedures.

For peer review only

For peer review only - http://bmjopen.bmj.com/site/about/guidelines.xhtml

METHODS AND ANALYSIS

We are using mixed methods methodology within this systematic review to answer complementary research questions within one study. In addition to answering questions of the effectiveness of self-consent interventions at increasing uptake of adolescent vaccination programmes, the systematic review will also synthesise qualitative research comprising the views of young people and relevant stakeholders to gain understanding of how self-consent procedures can be implemented effectively to increase uptake [9]. The findings from the qualitative and quantitative studies will be integrated to produce recommendations for future policy and practice [9].

This review protocol was prepared using the Preferred Reporting Items for Systematic Reviews and Meta-Analyses (PRISMA) Protocol guidelines [10] (Supplementary file 1) and has been registered with the International Prospective Register of Systematic Reviews (PROSPERO) (Registration number: CRD42017084509).

Search strategy

A comprehensive search strategy has been developed to capture all literature relevant to adolescent self-consent procedures for vaccination programmes by a reviewer (HB-F) experienced in undertaking systematic reviews in the proposed research field and discussed with members of the research team. The original search strategy developed for the Embase database has been adapted for each included database (see below) and comprises a combination of text words and the following medical subject headings (MeSH) indexing terms: 'child', 'adolescent', 'active immunization', 'immunization', 'immunization programs', 'mass immunization', 'revaccination', 'vaccination', 'diptheria vaccine', 'diptheria tetanus vaccine', 'diptheria pertussis tetanus', 'haemphilus influenzae type b vaccine', 'hepatitis b vaccine', 'meningcoccus vaccine', 'rubella vaccine', 'wart virus vaccine', 'papillomavirus vaccines', 'decision making', 'informed consent',

For peer review only - http://bmjopen.bmj.com/site/about/guidelines.xhtml

'parental consent', 'treatment refusal' (Table 1). Study design filters will not be applied as diverse study designs are eligible for inclusion.

Databases

To ensure all the relevant literature is captured, we will search the following ten databases from inception to January 2018 and re-run six months later (June 2018) to inform the wider research study as it progresses: Child Development & Adolescent Studies via EBSCOhost, Cochrane Central Register of Controlled Trials via The Cochrane Library, Cochrane Reviews via The Cochrane Library, Cumulative Index to Nursing and Allied Health Literature via EBSCOhost, Embase via Ovid, Health Technology Assessment Database, Medline via Ovid, PsycINFO via Ovid, Social Care Online via Social Care Institute for Excellence and Web of Science Core Collection: Social Sciences Citation Index and Conference Proceedings Citation Index- Science. All abstracts will be saved using Endnote X8.

Inclusion and exclusion criteria

Quantitative studies will be eligible if vaccine uptake following implementation of self-consent procedures is reported for young people aged between ten and 18 years [11]. Qualitative studies reporting the views and experiences of key stakeholder in relation adolescent self-consent procedures will also be included. Relevant stakeholders will vary with context but are likely to include young people, parents or primary care givers, healthcare professionals, policy makers, community leaders, and teachers.

We will include a range of study designs. To determine whether self-consent procedures can increase uptake of vaccination programmes, primary studies reporting parallel group randomised controlled trials, quasi-randomised trials, non-randomised controlled trials, controlled before and after studies, historically controlled studies, and retrospective or prospective cohort studies that include a control group will be eligible. Qualitative studies which use interviews, focus groups,

For peer review only - http://bmjopen.bmj.com/site/about/guidelines.xhtml

observations, or open-ended questions allowing free-text responses in questionnaires will be included to explore views and behaviours related to young people's self-consent for vaccination.

Conference abstracts, reviews, editorials, opinion pieces, dissertations, letters and books will only be included if they present original data. There will be no language or country of origin restriction imposed, and any relevant full text paper that is not written in English will be translated.

Study selection

Two reviewers will independently assess the titles and abstracts against the predefined eligibility criteria. Full-text publications of all potentially relevant articles will be retrieved and examined for relevance. Any disagreements arising will be resolved by discussion. The reference lists and bibliographies from relevant studies and systematic reviews will be hand-searched for additional primary studies not retrieved by the electronic search.

We will use the reference management software EndNote X8 to remove duplicates and sort exclusions and inclusions. The search strategy and study selection process will be documented using a PRISMA flow diagram [12].

Data extraction

At least two reviewers will independently extract data from selected studies using structured and standardised data extraction forms used in our previous qualitative and quantitative systematic reviews. In instances where multiple publications relate to the same study, these will be reported together. The following domains will be retrieved: study characteristics (authors, publication year, country, aim, study time period, study design, location, type of setting, data collection period, data collection method, sampling strategy, analysis), participant characteristics (participant age, sample size, vaccination status of participants, socioeconomic indicators, race/ethnicity, gender, and

religion) and study results (uptake of vaccine, views and behaviours related to self-consent procedures). Where possible, authors will be contacted for missing or incomplete data. Disagreements will be resolved through discussion.

Risk of bias and quality assessment

For eligible primary studies, quality assessment will be undertaken to illustrate potential sources of bias. As we anticipate the majority of eligible studies will be observational, studies will not automatically be excluded on the basis of 'low' quality assessment if they are considered to contribute relevant information. We propose using the Cochrane Collaboration's handbook for the assessment of risks of bias for systematic review of randomised controlled studies and quasi-randomised intervention studies [13], the Strengthening the Reporting of Observational Studies in Epidemiology (STROBE) appraisal tool for observational studies [14], and the Critical Appraisal Skills Programme criteria adapted for qualitative studies for evaluating qualitative research [15]. Quality assessment of primary studies will be undertaken independently by two reviewers and then an overall assessment of 'high', 'medium', 'low', or 'unclear' will be assigned and reported.

Data synthesis: Quantitative studies

We anticipate that the primary quantitative studies will be reported narratively as preliminary searches specifically related to HPV vaccination programmes indicated a lack of published studies and the likelihood of heterogeneity in relation to study design and reported outcomes. However, if sufficiently similar studies are captured we will consider combining individual study results through meta-analyses. To assess the heterogeneity between studies, we will use the Q-statistic and the I^2-statistics [16]. Evidence of heterogeneity will be classified as weak, moderate and strong for corresponding I^2 of 25%, 50% and 75% respectively. If heterogeneity between studies is classified as weak, analyses will comprise adjusted odds ratios (aORs) where available, with unadjusted odds ratios used if not reported. Analyses will be undertaken using the meta-analysis function [17]

available in Stata 15. We do not anticipate sufficient data being available to undertake sub-group analyses.

Data synthesis: Qualitative studies

The socio-ecological model [18] considers that behaviour is shaped by a complex interaction between factors operating at public policy, community, organisational, interpersonal, and intrapersonal levels. In a previous qualitative synthesis, we have shown that young women's access to the HPV vaccine is shaped by decisions at different levels of the socio-ecological model [5]. During the analysis, we will use the socio-ecological model to provide a framework for understanding how barriers and facilitators operating at different levels of the model can provide access to, or prevent, young people self-consenting in the context of vaccination programmes.

To analyse the qualitative data, the methodology for thematic synthesis reported by Thomas and Harden [19], assisted by the Framework method of qualitative data management [20], will be used. These methods are suited to studies with *a priori* aims and objectives. The overall purpose of the synthesis will be to 'pool' the results from individual primary studies by initially separating the findings, coding and interpreting the text, and then combining them through the identification of key themes across the studies as well as similarities and differences within those themes [21]. Thematic synthesis will be led by one reviewer reporting to the wider team about interpretation of the data as analysis progresses.

Familiarisation with the dataset will begin with reading the full papers. Pertinent sections of the text reported in each primary study will represent the basic units for analysis. Primary charts of the text will be constructed around key issues using the Framework Matrix within QSR NVivo10 software. For example, initial charts are likely to focus on 'barriers' and 'facilitators' to adolescent self-consent. The primary charts will be retained and revisited as required, but streamlined versions will be

For peer review only

produced as the process of coding, summarising and synthesising the data progresses. In subsequent charts, key terms and phrases will be retained while repetition within studies and extraneous text are removed. During this process, overarching themes will be identified, and differences or similarities explored within these emerging themes.

Data synthesis: Interrogation

The final stage of the analysis will aim to firstly comprise testing whether the recommendations developed from the qualitative studies have been addressed in evaluative studies retrieved for the review and, secondly, to examine whether interventions that match the recommendations result in higher uptake in vaccination [9].

ETHICS AND DISSEMINTATION

We will not seek ethical approval for this study because the secondary data to be collected cannot be linked to individuals. As far as we are aware, this will be the first systematic review to collate evidence in relation to adolescent self-consent procedures for vaccination programmes. The review comprises part of a larger study. The findings of this review will inform the larger study evaluating the practicality, acceptability and impact of new self-consent procedures for the schools-based HPV vaccination programme in the UK. Findings will also be used to make recommendations to improve self-consent procedures for young people in vaccination programmes. We anticipate the results of this study this may be of interest to national and international stakeholders interested in improving uptake in adolescent vaccination programmes.

FULL REFERENCES

1. World Health Organisation, Summary of WHO Position Papers - Recommendations for Routine Immunization, 2017. Available from - http://www.who.int/immunization/policy/Immunization_routine_table1.pdf?ua=1.

For peer review only

2. Public Health England, Green Book Chapter 11: The UK Immunisation Schedule, 2016. Available from - https://www.gov.uk/government/uploads/system/uploads/attachment_data/file/554298/Green_Book_Chapter_11.pdf.

3. Convention on the Rights of the Child. United Nations, Treaty Ser, 1989. **1577**.

4. World Health Organisation, Considerations regarding consent in vaccinating children and adolescents between 6 and 17 years old, 2014. Available from - http://www.who.int/immunization/programmes_systems/policies_strategies/consent_note_en.pdf.

5. Batista Ferrer, H., et al., Barriers and facilitators to HPV vaccination of young women in high-income countries: a qualitative systematic review and evidence synthesis. BMC Public Health, 2014. **14**(1): p. 700.

6. Batista Ferrer, H., Factors Influencing the Uptake of the Human Papillomavirus (HPV) Vaccination Programme. PhD Thesis. University of Bristol, November 2014.

7. Batista Ferrer, H., et al., Barriers and facilitators to uptake of the school-based HPV vaccination programme in an ethnically diverse group of young women. J Public Health, 2016. **38**(3): p. 569-577.

8. Audrey, S., et al., The practicality, acceptability and impact of self-consent procedures for the Human Papilloma Virus (HPV) vaccine in south-west England: a mixed methods study protocol. *Under review*.

9. Harden, A. and J. Thomas, Mixed methods and systematic reviews: examples and emerging issues. Mixed Methods Handbook, ed. A. Tashakkori and C. Teddlie. 2010, New York: Sage Publications.

10. Moher, D., et al., Preferred reporting items for systematic review and meta-analysis protocols (PRISMA-P) 2015 statement. Systematic Reviews, 2015. **4**(1): p. 1.

11. Young people's health--a challenge for society. Report of a WHO Study Group on young people and "Health for All by the Year 2000". World Health Organ Tech Rep Ser, 1986. **731**: p. 1-117.

12. Moher, D., et al., Preferred reporting items for systematic reviews and meta-analyses: The PRISMA statement. International Journal of Surgery, 2010. **8**(5): p. 336-341.

13. Higgins, J., Green, S., Cochrane Handbook for Systematic Reviews of Interventions, 2011. Available from - http://handbook.cochrane.org.

14. Von Elm, E., et al., The Strengthening the Reporting of Observational Studies in Epidemiology (STROBE) Statement: guidelines for reporting observational studies. International Journal of Surgery, 2014. **12**(12): p. 1495-1499.

15. Public Health Resource Unit, Critical Appraisal Skills Programme (CASP): appraisal tools, 1998. Available from - http://www.york.ac.uk/inst/crd/index_guidance.htm.

16. Higgins, J. and S. Thompson, Quantifying heterogeneity in a meta-analysis. Statistics in medicine, 2002. **21**(11): p. 1539-1558.

For peer review only

17. Sterne, J., M. Bradburn, and M. Egger, Meta–Analysis in Stata™, in Systematic Reviews in Health Care. 2008, BMJ Publishing Group. p. 347-369.

18. McLeroy, K., et al., An ecological perspective on health promotion programs. Health education quarterly, 1988. **15**(4): p. 351-377.

19. Thomas, J. and A. Harden, Methods for the thematic synthesis of qualitative research in systematic reviews. BMC Medical Research Methodology, 2008. **8**(1): p. 45.

20. Ritchie, J. Lewis J., ed. Qualitative research practice: a guide for social science students and researchers. 2003, Sage: London.

21. Estabrooks, C., P. Field, and J. Morse, Aggregating qualitative findings: an approach to theory development. Qualitative Health Research, 1994. **4**(4): p. 503-511.

LIST OF ABBREVIATIONS

HPV: Human Papillomavirus

WHO: World Health Organisation

UK: United Kingdom

USA: United States of America

PRISMA: Preferred Reporting Items for Systematic Reviews and Meta-Analyses Protocol

PROSPERO: Prospective Register of Systematic Reviews

MeSH: Medical Subject Headings

STROBE: Strengthening the Reporting of Observational Studies in Epidemiology

aOR: Adjusted Odds Ratio

OR: Odds Ratio

AUTHORS' CONTRIBUTIONS

All authors were involved in the conception and design of the research. SA is Principal Investigator; HB-F is study manager and lead researcher; JM and MH advise on systematic review methodology. HB-F wrote the first draft and all authors contributed to the final version of the manuscript.

For peer review only

FUNDING STATEMENT

This work is supported by the National Institute for Health Research for Patient Benefit (NIHR RfPB) programme (project number PB-PG-0416-20013). The work is also undertaken with the support of the NIHR Health Protection Research Unit in Evaluation of Interventions. The views and opinions expressed therein are those of the authors and do not necessarily reflect those of the NIHR RfPB Programme, the Department of Health, or Public Health England.

COMPETING INTERESTS STATEMENT

The authors declare there are no competing interests.

For peer review only

Table 1. Embase search strategy

1. child/
2. adolescent/
3. ("Young people#" OR "young person#" OR "young offender#" OR adolescent# OR adolescence OR youth# OR minor# OR teen OR teens OR teenage OR teenaged OR teenager# OR juvenile# OR pupil# OR boy# OR girl# OR underage# OR daughter# or son# (school AND dropout#) OR (school AND "drop out#") OR "school aged").mp.
4. active immunization/
5. immunization/
6. immunization programs/
7. mass immunization/
8. revaccination/
9. vaccination/
10. diphtheria vaccine/
11. diphtheria tetanus vaccine/
12. diphtheria pertussis tetanus Haemophilus influenzae type b vaccine/
13. hepatitis B vaccine/
14. meningococcus vaccine/
15. rubella vaccine/
16. wart virus vaccine/
17. Papillomavirus Vaccines/
18. (cervical cancer or diptheria or diphtheria or diphteria or DtaP or DTP or Hep B or hepatitis or HPV or measles or MenC or MenACWY or meningitis or Meningococcal or Neisseria meningitidis or papillomavirus or pertus* or rubella or rubeola or td?ipv or tetanus or wart virus or whoop*).tw.
19. (policy OR program*)
20. (immuniz* OR immunis* OR immunother* OR inoculat* OR innoculat* OR prophyla* OR revaccinat* OR vaccin*).mp.
21. Decision making/
22. Informed consent/
23. Parental consent/
24. Treatment refusal/
25. (assent* OR competen* OR decision-making OR decision making OR Gillick OR Fraser OR inform* consent OR mental capacity OR minor consent OR parent* consent OR permission* OR presume* consent OR treatment refusal OR self consent OR self-consent OR opt-out OR opt-in).mp.
26. 1 or 2 or 3
27. 4 or 5 or 6 or 7 or 8 or 9 or 10 or 11 or 12 or 13 or 14 or 15 or 16 or 17
28. 18 and 20
29. 19 and 20
30. 27 or 28 or 29
31. 21 or 22 or 23 or 24 or 25
32. 26 and 30 or 31

PRISMA-P 2015 Checklist

This checklist has been adapted for use with systematic review protocol submissions to BioMed Central journals from Table 3 in Moher D et al: Preferred reporting items for systematic review and meta-analysis protocols (PRISMA-P) 2015 statement. *Systematic Reviews* 2015 **4**:1

An Editorial from the Editors-in-Chief of *Systematic Reviews* details why this checklist was adapted - Moher D, Stewart L & Shekelle P: Implementing PRISMA-P: recommendations for prospective authors. *Systematic Reviews* 2016 **5**:15

Section/topic	#	Checklist item	Information reported		Line number(s)
			Yes	No	
**ADMINISTRATIVE INFORMATION**					
**Title**					
Identification	1a	Identify the report as a protocol of a systematic review	X	☐	1
Update	1b	If the protocol is for an update of a previous systematic review, identify as such	☐	X	
**Registration**	2	If registered, provide the name of the registry (e.g., PROSPERO) and registration number in the Abstract	X	☐	48, 124-25
**Authors**					
Contact	3a	Provide name, institutional affiliation, and e-mail address of all protocol authors; provide physical mailing address of corresponding author	X	☐	3-18
Contributions	3b	Describe contributions of protocol authors and identify the guarantor of the review	X	☐	345-348
**Amendments**	4	If the protocol represents an amendment of a previously completed or published protocol, identify as such and list changes; otherwise, state plan for documenting important protocol amendments	☐	X	
**Support**					
Sources	5a	Indicate sources of financial or other support for the review	X	☐	350-355
Sponsor	5b	Provide name for the review funder and/or sponsor	X	☐	350-355
Role of sponsor/funder	5c	Describe roles of funder(s), sponsor(s), and/or institution(s), if any, in developing the protocol	X	☐	350-355
**INTRODUCTION**					
**Rationale**	6	Describe the rationale for the review in the context of what is already known	X	☐	63-111

BioMed Central The Open Access Publisher

Section/topic	#	Checklist item	Information reported		Line number(s)
			Yes	No	
**Objectives**	7	Provide an explicit statement of the question(s) the review will address with reference to participants, interventions, comparators, and outcomes (PICO)	X		30-34, 108-111
**METHODS**					
**Eligibility criteria**	8	Specify the study characteristics (e.g., PICO, study design, setting, time frame) and report characteristics (e.g., years considered, language, publication status) to be used as criteria for eligibility for the review	X		140-168
**Information sources**	9	Describe all intended information sources (e.g., electronic databases, contact with study authors, trial registers, or other grey literature sources) with planned dates of coverage	X		140-148
**Search strategy**	10	Present draft of search strategy to be used for at least one electronic database, including planned limits, such that it could be repeated	X		360
STUDY RECORDS					
Data management	11a	Describe the mechanism(s) that will be used to manage records and data throughout the review	X		148, 177-178
Selection process	11b	State the process that will be used for selecting studies (e.g., two independent reviewers) through each phase of the review (i.e., screening, eligibility, and inclusion in meta-analysis)	X		170-203
Data collection process	11c	Describe planned method of extracting data from reports (e.g., piloting forms, done independently, in duplicate), any processes for obtaining and confirming data from investigators	X		181-191
**Data items**	12	List and define all variables for which data will be sought (e.g., PICO items, funding sources), any pre-planned data assumptions and simplifications	X		181-191
**Outcomes and prioritization**	13	List and define all outcomes for which data will be sought, including prioritization of main and additional outcomes, with rationale	X		189
**Risk of bias in individual studies**	14	Describe anticipated methods for assessing risk of bias of individual studies, including whether this will be done at the outcome or study level, or both; state how this information will be used in data synthesis	X		193-203
DATA					
**Synthesis**	15a	Describe criteria under which study data will be quantitatively synthesized	X		205-215
	15b	If data are appropriate for quantitative synthesis, describe planned summary measures, methods of handling data, and methods of combining data from studies, including any planned exploration of consistency (e.g., I^2, Kendall's tau)	X		205-215

BioMed Central The Open Access Publisher

For peer review only

3

Section/topic	#	Checklist item	Information reported		Line number(s)
			Yes	No	
	15c	Describe any proposed additional analyses (e.g., sensitivity or subgroup analyses, meta-regression)	X	☐	205-215
	15d	If quantitative synthesis is not appropriate, describe the type of summary planned	X	☐	206-208, 217-249
**Meta-bias(es)**	16	Specify any planned assessment of meta-bias(es) (e.g., publication bias across studies, selective reporting within studies)	☐	X	
**Confidence in cumulative evidence**	17	Describe how the strength of the body of evidence will be assessed (e.g., GRADE)	☐	X	

For peer review only

BioMed Central
The Open Access Publisher

BMJ Open

Adolescent self-consent for vaccinations: protocol for a mixed methods systematic review

Journal:	*BMJ Open*
Manuscript ID	bmjopen-2017-021335.R1
Article Type:	Protocol
Date Submitted by the Author:	26-Mar-2018
Complete List of Authors:	Batista, Harriet; University of Bristol, Population Health Sciences: Bristol Medical School<br>Hickman, M; University of Bristol, Population Health Sciences: Bristol Medical School<br>Macleod, John; University of Bristol, Population Health Sciences: Bristol Medical School<br>Audrey, Suzanne; University of Bristol, Population Health Sciences: Bristol Medical School
**Primary Subject Heading**:	Infectious diseases
Secondary Subject Heading:	Public health, Qualitative research
Keywords:	Self-consent, Vaccination, Systematic review, Mixed methods, Adolescents

SCHOLARONE™
Manuscripts

TITLE: Adolescent self-consent for vaccinations: protocol for a mixed methods systematic review

Dr Harriet Batista Ferrer, Population Health Sciences, Bristol Medical School, University of Bristol.

Professor Matthew Hickman, Population Health Sciences, Bristol Medical School, University of Bristol.

Professor John Macleod, Population Health Sciences, Bristol Medical School, University of Bristol.

Dr Suzanne Audrey, Population Health Sciences, Bristol Medical School, University of Bristol.

Corresponding author:

Dr Harriet Batista Ferrer

Senior Research Associate

Population Health Sciences, Bristol Medical School

University of Bristol

Canynge Hall

Whatley Road

Bristol BS8 2PS

Email: Harriet.Batista@bristol.ac.uk

Telephone: +44 (0) 117 928 7223

Word count: 2,219

Key words

Self-consent, Vaccination, Systematic review, Adolescents, Mixed Methods

For peer review only

ABSTRACT

Introduction: The recent global expansion of routine adolescent vaccination programmes has the potential to protect young people against the acquisition of infectious disease and improve their health. Although in many countries the legal framework supports young people to provide consent for medical interventions if they are considered competent, written parental consent can act as a barrier to uptake as it is frequently a condition of adolescent vaccination programmes. The aim of this systematic review protocol is to document the methods which will be used to identify, appraise and synthesise the available qualitative and quantitative evidence to address: (i) whether implementation of adolescent self-consent procedures can increase vaccination uptake, and; (ii) the barriers and facilitators to implementation of adolescent self-consent procedures.

Methods and analysis: Comprehensive search strategy of all relevant electronic databases for both qualitative and quantitative studies using predefined inclusion and exclusion criteria. At least two authors will independently review titles and abstracts, extract data and assess the methodological quality of eligible primary studies, resolving disagreements by consensus. Quantitative studies will be reported narratively and where possible pooled in a meta-analysis using a random-effects model. The findings of qualitative primary studies will be extracted, interpreted and synthesised to identify overarching themes as well as similarities and differences within those themes.

Ethics and dissemination: As this systematic review involves analysis of secondary data, the study does not require ethical approvals. We will use our findings to assess whether the evidence supports the hypothesis that self-consent procedures can increase coverage of adolescent vaccination programmes. We will identify barriers and facilitators to the implementation of adolescent self-consent for vaccination, and make recommendations for policy-makers and practitioners in relation to consent procedures within vaccination programmes for young people.

Systematic review registration: PROSPERO CRD42017084509

Word count: 284

For peer review only

Strengths and limitations of this study

- The mixed methods systematic review will answer complementary research questions about self-consent for adolescent vaccination programmes
- Robust systematic review methodology will be used to identify, appraise and synthesise the relevant qualitative and quantitative literature
- Lack of primary studies and heterogeneity of eligible studies in terms of study design, population and reporting may limit our ability to infer conclusions in relation to the research questions

For peer review only

INTRODUCTION

In recent years, the number of routine vaccinations recommended during adolescence have increased, and include vaccines that protect against tetanus, diphtheria, meningitis and human papillomavirus (HPV) acquisition [1, 2]. Provided sufficient coverage is achieved, the expansion of adolescent vaccination programmes may improve young people's health by protecting them from potentially life- threatening infectious diseases.

The introduction of new adolescent vaccination programmes is relevant to the debate about young people's capacity to provide consent to receive medical treatment. The United Nations Convention on the Rights of the Child recognises the right for all children and young people to participate in decision-making processes which involve them [3]. However, the World Health Organisation (WHO) has acknowledged difficulties over consent for vaccination of adolescents because of their age, and describes current practice through which countries are encouraged to adopt procedures that ensure parents have been informed and agreed to the vaccination [4].

In most countries, the legal framework for consent requires parental or guardian permission for young people aged below 18 years [4]. However, the age of consent for medical interventions, such as vaccination programmes, is lower in some countries. In the United Kingdom (UK), Canada and Sweden young women are legally able to override parental decisions if they are considered mature enough to make, and understand the consequences of, the decision to vaccinate. In Australia and the United States of America (USA) there are geographic variations of the age (12 to 17 years) that a young person can consent to be vaccinated. Despite young people being supported by the law to provide consent themselves, written parental consent is usually sought. In relation to the HPV vaccination programme, this has been shown to act as an important barrier preventing young women (usually aged 12 to 13 years) receiving the Human Papillomavirus (HPV) vaccine, with

For peer review only

implications for vaccination programme coverage [5, 6]. Furthermore, it is a barrier with potential to reinforce health inequalities since lack of written parental consent may also be related to lower socioeconomic status and some ethnic groups [5, 7].

To examine the issue of self-consent for the HPV vaccine in more detail, a mixed-methods study has been funded by the National Institute for Health Research (NIHR) Research for Patient Benefit Programme (RfPB) in England. The study is examining the practicality, acceptability and impact of implementing new self-consent procedures for the schools-based HPV vaccination in two local authorities in the south-west of England [8]. There are three elements to the study: statistical analyses of routine data to assess the impact of self-consent on overall uptake levels and in relation to socio-economic status, ethnicity and type of school; a process evaluation to examine the context, implementation and response to the new consent procedures, and; a systematic review of the evidence relating to self-consent for adolescent vaccines. The current protocol focusses on the systematic review which will run alongside, and inform, the other elements of the study.

An initial scoping search suggested a paucity of peer-reviewed evidence in relation to self-consent procedures for HPV vaccination programmes. Since issues relating to self-consent for the HPV vaccination are likely to be relevant for other vaccinations delivered during adolescence we widened the scope of the systematic review to identify and collate the evidence across all adolescent vaccination programmes. We chose to restrict to vaccination programmes, rather than include studies related to healthcare in general, to ensure the findings were relevant to the programme of research described above. Therefore, the aim of this mixed-methods systematic review is to identify, appraise and synthesise the available qualitative and quantitative literature to gain understanding as to: (i) whether implementation of adolescent self-consent procedures can increase vaccination uptake, and; (ii) the related barriers and facilitators to implementation of adolescent self-consent procedures.

For peer review only

METHODS AND ANALYSIS

We are using mixed methods methodology within this systematic review to answer complementary research questions within one study. In addition to answering questions of the effectiveness of self-consent interventions at increasing uptake of adolescent vaccination programmes, the systematic review will also synthesise qualitative research comprising the views of young people and relevant stakeholders to gain understanding of how self-consent procedures can be implemented effectively to increase uptake [9]. The findings from the qualitative and quantitative studies will be integrated to produce recommendations for future policy and practice [9].

This review protocol was prepared using the Preferred Reporting Items for Systematic Reviews and Meta-Analyses (PRISMA) Protocol guidelines [10] (Supplementary file 1) and has been registered with the International Prospective Register of Systematic Reviews (PROSPERO) (Registration number: CRD42017084509).

Search strategy

A comprehensive search strategy has been developed to capture all literature relevant to adolescent self-consent procedures for vaccination programmes by a reviewer (HB-F) experienced in undertaking systematic reviews in the proposed research field and discussed with members of the research team. The original search strategy developed for the Embase database has been adapted for each included database (see below) and comprises a combination of text words and the following medical subject headings (MeSH) indexing terms: ‘child’, ‘adolescent’, ‘active immunization’, ‘immunization’, ‘immunization programs’, ‘mass immunization’, ‘revaccination’, ‘vaccination’, ‘diptheria vaccine’, ‘diptheria tetanus vaccine’, ‘diptheria pertussis tetanus’, ‘haemphilus influenzae type b vaccine’, ‘hepatitis b vaccine’, ‘meningcoccus vaccine’, ‘rubella vaccine’, ‘wart virus vaccine’, ‘papillomavirus vaccines’, ‘decision making’, ‘informed consent’,

For peer review only - http://bmjopen.bmj.com/site/about/guidelines.xhtml

'parental consent', 'treatment refusal' (Table 1). Study design filters or restrictions by setting will not be applied as the study aims to be inclusive in relation to study design and settings eligible for inclusion.

Databases

To ensure all the relevant literature is captured, we will search the following ten databases from inception to January 2018 and re-run six months later (June 2018) to inform the wider research study as it progresses: Child Development & Adolescent Studies via EBSCOhost, Cochrane Central Register of Controlled Trials via The Cochrane Library, Cochrane Reviews via The Cochrane Library, Cumulative Index to Nursing and Allied Health Literature via EBSCOhost, Embase via Ovid, Health Technology Assessment Database, Medline via Ovid, PsycINFO via Ovid, Social Care Online via Social Care Institute for Excellence and Web of Science Core Collection: Social Sciences Citation Index and Conference Proceedings Citation Index- Science. All abstracts will be saved using Endnote X8.

Inclusion and exclusion criteria

Quantitative studies will be eligible if vaccine uptake following implementation of self-consent procedures is reported for young people aged between ten and 18 years [11]. Qualitative studies reporting the views and experiences of key stakeholders in relation adolescent self-consent procedures will also be included. Studies related to consent procedures solely targeting parents of adolescents, or early childhood and adult vaccination programmes will not be eligible for inclusion. Relevant stakeholders will vary with context but are likely to include young people, parents or primary care givers, healthcare professionals, policy makers, community leaders and teachers.

We will include a range of study designs. To determine whether self-consent procedures can increase uptake of vaccination programmes, primary studies reporting parallel group randomised controlled trials, quasi-randomised trials, non-randomised controlled trials, controlled before and

For peer review only - http://bmjopen.bmj.com/site/about/guidelines.xhtml

after studies, historically controlled studies, and retrospective or prospective cohort studies that include a control group will be eligible. Qualitative studies which use interviews, focus groups, observations, or open-ended questions allowing free-text responses in questionnaires will be included to explore views and behaviours related to young people's self-consent for vaccination.

Conference abstracts, reviews, editorials, opinion pieces, dissertations, letters and books will only be included if they present original data. There will be no language or country of origin restriction imposed, and any relevant full text paper that is not written in English will be translated.

Study selection

Two reviewers will independently assess the titles and abstracts against the predefined eligibility criteria. Full-text publications of all potentially relevant articles will be retrieved and examined for relevance. Any disagreements arising will be resolved by discussion. The reference lists and bibliographies from relevant studies and systematic reviews will be hand-searched for additional primary studies not retrieved by the electronic search.

We will use the reference management software EndNote X8 to remove duplicates and sort exclusions and inclusions. The search strategy and study selection process will be documented using a PRISMA flow diagram [12].

Data extraction

At least two reviewers will independently extract data from selected studies using structured and standardised data extraction forms used in our previous qualitative and quantitative systematic reviews. In instances where multiple publications relate to the same study, these will be reported together. The following domains will be retrieved: study characteristics (authors, publication year, country, aim, study time period, study design, location, type of setting, data collection period, data

collection method, sampling strategy, analysis, participant characteristics (participant age, sample size, vaccination status of participants, socioeconomic indicators, race/ethnicity, gender, and religion) and study results (uptake of vaccine, psychological outcomes, healthcare service use, incidence of vaccine preventable disease, views and behaviours related to self-consent procedures, authors' reported conflicts of interest and study funding sources). We will also record data relating to the possible harms resulting from self-consent procedures (e.g. conflict with parents, healthcare professional anxiety). Where possible, authors will be contacted for missing or incomplete data. Disagreements will be resolved through discussion.

Risk of bias and quality assessment

For eligible primary studies, quality assessment will be undertaken to illustrate potential sources of bias. As we anticipate the majority of eligible studies will be observational, studies will not automatically be excluded on the basis of 'low' quality assessment if they are considered to contribute relevant information. We propose using: the Cochrane Collaboration's handbook for the assessment of risks of bias for systematic review of randomised controlled studies and quasi-randomised intervention studies [13] Risk Of Bias in Non Randomised Studies of Interventions (ROBINS-I) [14] ;, the NIH Quality Assessment Tool for Observational Cohort and Cross-Sectional Studies [15], and; the Critical Appraisal Skills Programme criteria adapted for qualitative studies for evaluating qualitative research [16]. Quality assessment of primary studies will be undertaken independently by two reviewers and recorded in an excel spreadsheet. An overall assessment of 'high', 'medium', 'low', or 'unclear' will be assigned and reported.

Data synthesis: Quantitative studies

We anticipate that the primary quantitative studies will be reported narratively as preliminary searches specifically related to HPV vaccination programmes indicated a lack of published studies and the likelihood of heterogeneity in relation to study design and reported outcomes. However, if

sufficiently similar studies are captured we will consider combining individual study results through meta-analyses. To assess the heterogeneity between studies, we will use the Q-statistic and the I^2-statistics [17]. Evidence of heterogeneity will be classified as weak, moderate and strong for corresponding I^2 of 25%, 50% and 75% respectively. If heterogeneity between studies is classified as weak, analyses will comprise adjusted odds ratios (aORs) where available, with unadjusted odds ratios used if not reported. Analyses will be undertaken using the meta-analysis function [18] available in Stata 15. We do not anticipate sufficient data being available to undertake sub-group analyses. However, if sufficient data were reported we propose two sub-group analyses to compare impact of self-consent procedures by: (i) setting (healthcare vs. school) and (ii) age of participants (less than 14 years old vs. 14 years and greater).

Data synthesis: Qualitative studies

The socio-ecological model [19] considers that behaviour is shaped by a complex interaction between factors operating at public policy, community, organisational, interpersonal and intrapersonal levels. In a previous qualitative synthesis, we have shown that young women's access to the HPV vaccine is shaped by decisions at different levels of the socio-ecological model [5]. During the analysis, we will use the socio-ecological model to provide a framework for understanding how barriers and facilitators operating at different levels of the model can provide access to, or prevent, young people self-consenting in the context of vaccination programmes.

To analyse the qualitative data, the methodology for thematic synthesis reported by Thomas and Harden [20], assisted by the Framework method of qualitative data management [21], will be used. These methods are suited to studies with *a priori* aims and objectives. The overall purpose of the synthesis will be to 'pool' the results from individual primary studies by initially separating the findings, coding and interpreting the text, and then combining them through the identification of key themes across the studies as well as similarities and differences within those themes [22]. Thematic

For peer review only - http://bmjopen.bmj.com/site/about/guidelines.xhtml

synthesis will be led by one reviewer reporting to the wider team about interpretation of the data as analysis progresses.

Familiarisation with the dataset will begin with reading the full papers. Pertinent sections of the text reported in each primary study will represent the basic units for analysis. Primary charts of the text will be constructed around key issues using the Framework Matrix within QSR NVivo10 software. For example, initial charts are likely to focus on 'barriers' and 'facilitators' to adolescent self-consent. The primary charts will be retained and revisited as required. Streamlined versions will be produced as the process of coding, summarising and synthesising the data progresses. In subsequent charts, key terms and phrases will be retained while repetition within studies and extraneous text are removed. During this process, overarching themes will be identified, and differences or similarities explored within these emerging themes.

Data synthesis: Interrogation

The final stage of the analysis will aim, firstly, to test whether the recommendations developed from the qualitative studies have been addressed in evaluative studies retrieved for the review and, secondly, to examine whether interventions that match the recommendations result in higher uptake in vaccination [9].

Patient and public involvement

The Bristol Young People's Advisory Group (YPAG) comprises young people aged ten to 17 years who are interested in healthcare and research. They meet regularly to help researchers with their projects and have been consulted about the design of the wider study and participant materials. They will also be invited to an event at the end of the study to consider findings and recommendations with the young people, parents, immunisation nurses and school staff involved in the study.

For peer review only

ETHICS AND DISSEMINTATION

We will not seek ethical approval for this study because the secondary data to be collected cannot be linked to individuals. As far as we are aware, this will be the first systematic review to collate evidence in relation to adolescent self-consent procedures for vaccination programmes. The review comprises part of a larger study. The findings of this review will inform the larger study evaluating the practicality, acceptability and impact of new self-consent procedures for the schools-based HPV vaccination programme in the UK. Findings will also be used to make recommendations to improve self-consent procedures for young people in vaccination programmes. We anticipate the results of this study this may be of interest to national and international stakeholders interested in improving uptake in adolescent vaccination programmes.

FULL REFERENCES

1. World Health Organisation, Summary of WHO Position Papers - Recommendations for Routine Immunization, 2017. Available from - http://www.who.int/immunization/policy/Immunization_routine_table1.pdf?ua=1.

2. Public Health England, Green Book Chapter 11: The UK Immunisation Schedule, 2016. Available from - https://www.gov.uk/government/uploads/system/uploads/attachment_data/file/554298/Green_Book_Chapter_11.pdf.

3. Convention on the Rights of the Child. United Nations, Treaty Ser, 1989. **1577**.

4. World Health Organisation, Considerations regarding consent in vaccinating children and adolescents between 6 and 17 years old, 2014. Available from - http://www.who.int/immunization/programmes_systems/policies_strategies/consent_note_en.pdf.

5. Batista Ferrer, H., et al., Barriers and facilitators to HPV vaccination of young women in high-income countries: a qualitative systematic review and evidence synthesis. BMC Public Health, 2014. **14**(1): p. 700.

6. Batista Ferrer, H., Factors Influencing the Uptake of the Human Papillomavirus (HPV) Vaccination Programme. PhD Thesis. University of Bristol, November 2014.

For peer review only

7. Batista Ferrer, H., et al., Barriers and facilitators to uptake of the school-based HPV vaccination programme in an ethnically diverse group of young women. J Public Health (Oxf), 2016. **38**(3): p. 569-577.

8. Audrey, S., et al., Impact and acceptability of self-consent procedures for the school-based human papillomavirus vaccine: a mixed-methods study protocol. BMJ Open, 2018. **8**(3).

9. Harden, A. and J. Thomas, Mixed methods and systematic reviews: examples and emerging issues. Mixed Methods Handbook, ed. A. Tashakkori and C. Teddlie. 2010, New York: Sage Publications.

10. Moher, D., et al., Preferred reporting items for systematic review and meta-analysis protocols (PRISMA-P) 2015 statement. Systematic Reviews, 2015. **4**(1): p. 1.

11. Young people's health--a challenge for society. Report of a WHO Study Group on young people and "Health for All by the Year 2000". World Health Organ Tech Rep Ser, 1986. **731**: p. 1-117.

12. Moher, D., et al., Preferred reporting items for systematic reviews and meta-analyses: The PRISMA statement. International Journal of Surgery, 2010. **8**(5): p. 336-341.

13. Higgins J, Green, S., Cochrane Handbook for Systematic Reviews of Interventions, 2011. Available from - http://handbook.cochrane.org.

14. Sterne, J.A., et al., ROBINS-I: a tool for assessing risk of bias in non-randomised studies of interventions. BMJ, 2016. **355**.

15. National Institute for Health Quality Assessment Tool for Observational Cohort and Cross-Sectional Studies, 2017. Available from - https://www.nhlbi.nih.gov/health-topics/study-quality-assessment-tools.

16. Public Health Resource Unit, Critical Appraisal Skills Programme (CASP): appraisal tools, 1998. Available from - http://www.york.ac.uk/inst/crd/index_guidance.htm.

17. Higgins, J. and S.G. Thompson, Quantifying heterogeneity in a meta-analysis. Statistics in medicine, 2002. **21**(11): p. 1539-1558.

18. Sterne, J., M.J. Bradburn, and M. Egger, Meta–Analysis in Stata™, in Systematic Reviews in Health Care. 2008, BMJ Publishing Group. p. 347-369.

19. McLeroy, K.R., et al., An ecological perspective on health promotion programs. Health education quarterly, 1988. **15**(4): p. 351-377.

20. Thomas, J. and A. Harden, Methods for the thematic synthesis of qualitative research in systematic reviews. BMC Medical Research Methodology, 2008. **8**(1): p. 45.

21. Ritchie J, Lewis .J., ed. Qualitative research practice: a guide for social science students and researchers. 2003, Sage: London.

22. Estabrooks, C.A., P.A. Field, and J.M. Morse, Aggregating qualitative findings: an approach to theory development. Qualitative Health Research, 1994. **4**(4): p. 503-511.

For peer review only

LIST OF ABBREVIATIONS

HPV: Human Papillomavirus

WHO: World Health Organisation

UK: United Kingdom

USA: United States of America

PRISMA: Preferred Reporting Items for Systematic Reviews and Meta-Analyses Protocol

PROSPERO: Prospective Register of Systematic Reviews

MeSH: Medical Subject Headings

STROBE: Strengthening the Reporting of Observational Studies in Epidemiology

aOR: Adjusted Odds Ratio

OR: Odds Ratio

YPAG: Young Person's Advisory Group

AUTHORS' CONTRIBUTIONS

All authors were involved in the conception and design of the research. SA is Principal Investigator; HB-F is study manager and lead researcher; JM and MH advise on systematic review methodology. HB-F wrote the first draft and all authors contributed to the final version of the manuscript.

FUNDING STATEMENT

This work is supported by the National Institute for Health Research for Patient Benefit (NIHR RfPB) programme (project number PB-PG-0416-20013). The work is also undertaken with the support of the NIHR Health Protection Research Unit in Evaluation of Interventions. The work was also undertaken with the support of The Centre for the Development and Evaluation of Complex Interventions for Public Health Improvement (DECIPHer), a UKCRC Public Health Research Centre of Excellence. Joint funding (MR/KO232331/1) from the British Heart Foundation, Cancer Research UK,

For peer review only

Economic and Social Research Council, Medical Research Council, the Welsh Government and the Wellcome Trust, under the auspices of the UK Clinical Research Collaboration, is gratefully acknowledged. The views and opinions expressed therein are those of the authors and do not necessarily reflect those of the NIHR RfPB Programme, the Department of Health, or Public Health England.

COMPETING INTERESTS STATEMENT

The authors declare there are no competing interests.

For peer review only

Table 1. Embase search strategy

1. child/
2. adolescent/
3. ("Young people#" OR "young person#" OR "young offender#" OR adolescent# OR adolescence OR youth# OR minor# OR teen OR teens OR teenage OR teenaged OR teenager# OR juvenile# OR pupil# OR boy# OR girl# OR underage# OR daughter# or son# (school AND dropout#) OR (school AND "drop out#") OR "school aged").mp.
4. active immunization/
5. immunization/
6. immunization programs/
7. mass immunization/
8. revaccination/
9. vaccination/
10. diphtheria vaccine/
11. diphtheria tetanus vaccine/
12. diphtheria pertussis tetanus Haemophilus influenzae type b vaccine/
13. hepatitis B vaccine/
14. meningococcus vaccine/
15. rubella vaccine/
16. wart virus vaccine/
17. Papillomavirus Vaccines/
18. (cervical cancer or diptheria or diphtheria or diphteria or DtaP or DTP or Hep B or hepatitis or HPV or measles or MenC or MenACWY or meningitis or Meningococcal or Neisseria meningitidis or papillomavirus or pertus* or rubella or rubeola or td?ipv or tetanus or wart virus or whoop*).tw.
19. (policy OR program*)
20. (immuniz* OR immunis* OR immunother* OR inoculat* OR innoculat* OR prophyla* OR revaccinat* OR vaccin*).mp.
21. Decision making/
22. Informed consent/
23. Parental consent/
24. Treatment refusal/
25. (assent* OR competen* OR decision-making OR decision making OR Gillick OR Fraser OR inform* consent OR mental capacity OR minor consent OR parent* consent OR permission* OR presume* consent OR treatment refusal OR self consent OR self-consent OR opt-out OR opt-in).mp.
26. 1 or 2 or 3
27. 4 or 5 or 6 or 7 or 8 or 9 or 10 or 11 or 12 or 13 or 14 or 15 or 16 or 17
28. 18 and 20
29. 19 and 20
30. 27 or 28 or 29
31. 21 or 22 or 23 or 24 or 25
32. 26 and 30 or 31

For peer review only - http://bmjopen.bmj.com/site/about/guidelines.xhtml

PRISMA-P 2015 Checklist

This checklist has been adapted for use with systematic review protocol submissions to BioMed Central journals from Table 3 in Moher D et al: Preferred reporting items for systematic review and meta-analysis protocols (PRISMA-P) 2015 statement. *Systematic Reviews* 2015 4:1

An Editorial from the Editors-in-Chief of *Systematic Reviews* details why this checklist was adapted - Moher D, Stewart L & Shekelle P: Implementing PRISMA-P: recommendations for prospective authors. *Systematic Reviews* 2016 5:15

Section/topic	#	Checklist item	Information reported		Line number(s)
			Yes	No	
**ADMINISTRATIVE INFORMATION**					
**Title**					
Identification	1a	Identify the report as a protocol of a systematic review	X	☐	1
Update	1b	If the protocol is for an update of a previous systematic review, identify as such	☐	X	
**Registration**	2	If registered, provide the name of the registry (e.g., PROSPERO) and registration number in the Abstract	X	☐	48, 120-21
**Authors**					
Contact	3a	Provide name, institutional affiliation, and e-mail address of all protocol authors; provide physical mailing address of corresponding author	X	☐	3-18
Contributions	3b	Describe contributions of protocol authors and identify the guarantor of the review	X	☐	362-365
**Amendments**	4	If the protocol represents an amendment of a previously completed or published protocol, identify as such and list changes; otherwise, state plan for documenting important protocol amendments	☐	X	
**Support**					
Sources	5a	Indicate sources of financial or other support for the review	X	☐	367-377
Sponsor	5b	Provide name for the review funder and/or sponsor	X	☐	367-377
Role of sponsor/funder	5c	Describe roles of funder(s), sponsor(s), and/or institution(s), if any, in developing the protocol	X	☐	367-377
**INTRODUCTION**					
**Rationale**	6	Describe the rationale for the review in the context of what is already known	X	☐	59-107

For peer review only

BioMed Central The Open Access Publisher

Section/topic	#	Checklist item	Information reported Yes	Information reported No	Line number(s)
**Objectives**	7	Provide an explicit statement of the question(s) the review will address with reference to participants, interventions, comparators, and outcomes (PICO)	X	☐	32-34, 104-107
**METHODS**					
**Eligibility criteria**	8	Specify the study characteristics (e.g., PICO, study design, setting, time frame) and report characteristics (e.g., years considered, language, publication status) to be used as criteria for eligibility for the review	X	☐	147-166
**Information sources**	9	Describe all intended information sources (e.g., electronic databases, contact with study authors, trial registers, or other grey literature sources) with planned dates of coverage	X	☐	147-166
**Search strategy**	10	Present draft of search strategy to be used for at least one electronic database, including planned limits, such that it could be repeated	X	☐	382
STUDY RECORDS					
Data management	11a	Describe the mechanism(s) that will be used to manage records and data throughout the review	X	☐	145, 175-176
Selection process	11b	State the process that will be used for selecting studies (e.g., two independent reviewers) through each phase of the review (i.e., screening, eligibility, and inclusion in meta-analysis)	X	☐	168-177
Data collection process	11c	Describe planned method of extracting data from reports (e.g., piloting forms, done independently, in duplicate), any processes for obtaining and confirming data from investigators	X	☐	179-192
**Data items**	12	List and define all variables for which data will be sought (e.g., PICO items, funding sources), any pre-planned data assumptions and simplifications	X	☐	183-192
**Outcomes and prioritization**	13	List and define all outcomes for which data will be sought, including prioritization of main and additional outcomes, with rationale	X	☐	183-192
**Risk of bias in individual studies**	14	Describe anticipated methods for assessing risk of bias of individual studies, including whether this will be done at the outcome or study level, or both; state how this information will be used in data synthesis	X	☐	194-205
DATA					
**Synthesis**	15a	Describe criteria under which study data will be quantitatively synthesized	X	☐	207-220
	15b	If data are appropriate for quantitative synthesis, describe planned summary measures, methods of handling data, and methods of combining data from studies, including any planned exploration of consistency (e.g., I^2, Kendall's tau)	X	☐	207-220

BioMed Central The Open Access Publisher

Section/topic	#	Checklist item	Information reported Yes	Information reported No	Line number(s)
	15c	Describe any proposed additional analyses (e.g., sensitivity or subgroup analyses, meta-regression)	X	☐	217-220
	15d	If quantitative synthesis is not appropriate, describe the type of summary planned	X	☐	208-210, 222-254
**Meta-bias(es)**	16	Specify any planned assessment of meta-bias(es) (e.g., publication bias across studies, selective reporting within studies)	☐	X	
**Confidence in cumulative evidence**	17	Describe how the strength of the body of evidence will be assessed (e.g., GRADE)	☐	X	

For peer review only

BioMed Central The Open Access Publisher

BMJ Open

Adolescent self-consent for vaccinations: protocol for a mixed methods systematic review

Journal:	*BMJ Open*
Manuscript ID	bmjopen-2017-021335.R2
Article Type:	Protocol
Date Submitted by the Author:	19-Apr-2018
Complete List of Authors:	Batista, Harriet; University of Bristol, Population Health Sciences: Bristol Medical School<br>Hickman, M; University of Bristol, Population Health Sciences: Bristol Medical School<br>Macleod, John; University of Bristol, Population Health Sciences: Bristol Medical School<br>Audrey, Suzanne; University of Bristol, Population Health Sciences: Bristol Medical School
**Primary Subject Heading**:	Infectious diseases
Secondary Subject Heading:	Public health, Qualitative research
Keywords:	Self-consent, Vaccination, Systematic review, Mixed methods, Adolescents

SCHOLARONE™
Manuscripts

For peer review only - http://bmjopen.bmj.com/site/about/guidelines.xhtml

TITLE: Adolescent self-consent for vaccinations: protocol for a mixed methods systematic review

Dr Harriet Batista Ferrer, Population Health Sciences, Bristol Medical School, University of Bristol.

Professor Matthew Hickman, Population Health Sciences, Bristol Medical School, University of Bristol.

Professor John Macleod, Population Health Sciences, Bristol Medical School, University of Bristol.

Dr Suzanne Audrey, Population Health Sciences, Bristol Medical School, University of Bristol.

Corresponding author:

Dr Harriet Batista Ferrer

Senior Research Associate

Population Health Sciences, Bristol Medical School

University of Bristol

Canynge Hall

Whatley Road

Bristol BS8 2PS

Email: Harriet.Batista@bristol.ac.uk

Telephone: +44 (0) 117 928 7223

Word count: 2,219

Key words

Self-consent, Vaccination, Systematic review, Adolescents, Mixed Methods

For peer review only

ABSTRACT

Introduction: The recent global expansion of routine adolescent vaccination programmes has the potential to protect young people against the acquisition of infectious disease and improve their health. Although in many countries the legal framework supports young people to provide consent for medical interventions if they are considered competent, written parental consent can act as a barrier to uptake as it is frequently a condition of adolescent vaccination programmes. The aim of this systematic review protocol is to document the methods which will be used to identify, appraise and synthesise the available qualitative and quantitative evidence to address: (i) whether implementation of adolescent self-consent procedures can increase vaccination uptake, and; (ii) the barriers and facilitators to implementation of adolescent self-consent procedures.

Methods and analysis: Comprehensive search strategy of all relevant electronic databases for both qualitative and quantitative studies using predefined inclusion and exclusion criteria. At least two authors will independently review titles and abstracts, extract data and assess the methodological quality of eligible primary studies, resolving disagreements by consensus. Quantitative studies will be reported narratively and where possible pooled in a meta-analysis using a random-effects model. The findings of qualitative primary studies will be extracted, interpreted and synthesised to identify overarching themes as well as similarities and differences within those themes.

Ethics and dissemination: As this systematic review involves analysis of secondary data, the study does not require ethical approvals. We will use our findings to assess whether the evidence supports the hypothesis that self-consent procedures can increase coverage of adolescent vaccination programmes. We will identify barriers and facilitators to the implementation of adolescent self-consent for vaccination, and make recommendations for policy-makers and practitioners in relation to consent procedures within vaccination programmes for young people.

Systematic review registration: PROSPERO CRD42017084509

Word count: 284

For peer review only

Strengths and limitations of this study

- The mixed methods systematic review will answer complementary research questions about self-consent for adolescent vaccination programmes
- Robust systematic review methodology will be used to identify, appraise and synthesise the relevant qualitative and quantitative literature
- Lack of primary studies and heterogeneity of eligible studies in terms of study design, population and reporting may limit our ability to infer conclusions in relation to the research questions

For peer review only

INTRODUCTION

In recent years, the number of routine vaccinations recommended during adolescence have increased, and include vaccines that protect against tetanus, diphtheria, meningitis and human papillomavirus (HPV) acquisition [1, 2]. Provided sufficient coverage is achieved, the expansion of adolescent vaccination programmes may improve young people's health by protecting them from potentially life- threatening infectious diseases.

The introduction of new adolescent vaccination programmes is relevant to the debate about young people's capacity to provide consent to receive medical treatment. The United Nations Convention on the Rights of the Child recognises the right for all children and young people to participate in decision-making processes which involve them [3]. However, the World Health Organisation (WHO) has acknowledged difficulties over consent for vaccination of adolescents because of their age, and describes current practice through which countries are encouraged to adopt procedures that ensure parents have been informed and agreed to the vaccination [4].

In most countries, the legal framework for consent requires parental or guardian permission for young people aged below 18 years [4]. However, the age of consent for medical interventions, such as vaccination programmes, is lower in some countries. In the United Kingdom (UK), Canada and Sweden young women are legally able to override parental decisions if they are considered mature enough to make, and understand the consequences of, the decision to vaccinate. In Australia and the United States of America (USA) there are geographic variations of the age (12 to 17 years) that a young person can consent to be vaccinated. Despite young people being supported by the law to provide consent themselves, written parental consent is usually sought. In relation to the HPV vaccination programme, this has been shown to act as an important barrier preventing young women (usually aged 12 to 13 years) receiving the Human Papillomavirus (HPV) vaccine, with

For peer review only

implications for vaccination programme coverage [5, 6]. Furthermore, it is a barrier with potential to reinforce health inequalities since lack of written parental consent may also be related to lower socioeconomic status and some ethnic groups [5, 7].

To examine the issue of self-consent for the HPV vaccine in more detail, a mixed-methods study has been funded by the National Institute for Health Research (NIHR) Research for Patient Benefit Programme (RfPB) in England. The study is examining the practicality, acceptability and impact of implementing new self-consent procedures for the schools-based HPV vaccination in two local authorities in the south-west of England [8]. There are three elements to the study: statistical analyses of routine data to assess the impact of self-consent on overall uptake levels and in relation to socio-economic status, ethnicity and type of school; a process evaluation to examine the context, implementation and response to the new consent procedures, and; a systematic review of the evidence relating to self-consent for adolescent vaccines. The current protocol focusses on the systematic review which will run alongside, and inform, the other elements of the study.

An initial scoping search suggested a paucity of peer-reviewed evidence in relation to self-consent procedures for HPV vaccination programmes. Since issues relating to self-consent for the HPV vaccination are likely to be relevant for other vaccinations delivered during adolescence we widened the scope of the systematic review to identify and collate the evidence across all adolescent vaccination programmes. We chose to restrict to vaccination programmes, rather than include studies related to healthcare in general, to ensure the findings were relevant to the programme of research described above. Therefore, the aim of this mixed-methods systematic review is to identify, appraise and synthesise the available qualitative and quantitative literature to gain understanding as to: (i) whether implementation of adolescent self-consent procedures can increase vaccination uptake, and; (ii) the related barriers and facilitators to implementation of adolescent self-consent procedures.

For peer review only

METHODS AND ANALYSIS

We are using mixed methods methodology within this systematic review to answer complementary research questions within one study. In addition to answering questions of the effectiveness of self-consent interventions at increasing uptake of adolescent vaccination programmes, the systematic review will also synthesise qualitative research comprising the views of young people and relevant stakeholders to gain understanding of how self-consent procedures can be implemented effectively to increase uptake [9]. The findings from the qualitative and quantitative studies will be integrated to produce recommendations for future policy and practice [9].

This review protocol was prepared using the Preferred Reporting Items for Systematic Reviews and Meta-Analyses (PRISMA) Protocol guidelines [10] (Supplementary file 1) and has been registered with the International Prospective Register of Systematic Reviews (PROSPERO) (Registration number: CRD42017084509).

Search strategy

A comprehensive search strategy has been developed to capture all literature relevant to adolescent self-consent procedures for vaccination programmes by a reviewer (HB-F) experienced in undertaking systematic reviews in the proposed research field and discussed with members of the research team. The original search strategy developed for the Embase database has been adapted for each included database (see below) and comprises a combination of text words and the following medical subject headings (MeSH) indexing terms: 'child', 'adolescent', 'active immunization', 'immunization', 'immunization programs', 'mass immunization', 'revaccination', 'vaccination', 'diptheria vaccine', 'diptheria tetanus vaccine', 'diptheria pertussis tetanus', 'haemphilus influenzae type b vaccine', 'hepatitis b vaccine', 'meningcoccus vaccine', 'rubella vaccine', 'wart virus vaccine', 'papillomavirus vaccines', 'decision making', 'informed consent',

For peer review only - http://bmjopen.bmj.com/site/about/guidelines.xhtml

'parental consent', 'treatment refusal' (Table 1). Study design filters or restrictions by setting will not be applied as the study aims to be inclusive in relation to study design and settings eligible for inclusion.

Databases

To ensure all the relevant literature is captured, we will search the following ten databases from inception to January 2018 and re-run six months later (June 2018) to inform the wider research study as it progresses: Child Development & Adolescent Studies via EBSCOhost, Cochrane Central Register of Controlled Trials via The Cochrane Library, Cochrane Reviews via The Cochrane Library, Cumulative Index to Nursing and Allied Health Literature via EBSCOhost, Embase via Ovid, Health Technology Assessment Database, Medline via Ovid, PsycINFO via Ovid, Social Care Online via Social Care Institute for Excellence and Web of Science Core Collection: Social Sciences Citation Index and Conference Proceedings Citation Index- Science. All abstracts will be saved using Endnote X8.

Inclusion and exclusion criteria

Quantitative studies will be eligible if vaccine uptake following implementation of self-consent procedures is reported for young people aged between ten and 18 years [11]. Qualitative studies reporting the views and experiences of key stakeholders in relation adolescent self-consent procedures will also be included. Studies related to consent procedures solely targeting parents of adolescents, or early childhood and adult vaccination programmes will not be eligible for inclusion. Relevant stakeholders will vary with context but are likely to include young people, parents or primary care givers, healthcare professionals, policy makers, community leaders and teachers.

We will include a range of study designs. To determine whether self-consent procedures can increase uptake of vaccination programmes, primary studies reporting parallel group randomised controlled trials, quasi-randomised trials, non-randomised controlled trials, controlled before and

after studies, historically controlled studies, and retrospective or prospective cohort studies that include a control group will be eligible. Qualitative studies which use interviews, focus groups, observations, or open-ended questions allowing free-text responses in questionnaires will be included to explore views and behaviours related to young people's self-consent for vaccination.

Conference abstracts, reviews, editorials, opinion pieces, dissertations, letters and books will only be included if they present original data. There will be no language or country of origin restriction imposed, and any relevant full text paper that is not written in English will be translated.

Study selection

Two reviewers will independently assess the titles and abstracts against the predefined eligibility criteria. Full-text publications of all potentially relevant articles will be retrieved and examined for relevance. Any disagreements arising will be resolved by discussion. The reference lists and bibliographies from relevant studies and systematic reviews will be hand-searched for additional primary studies not retrieved by the electronic search.

We will use the reference management software EndNote X8 to remove duplicates and sort exclusions and inclusions. The search strategy and study selection process will be documented using a PRISMA flow diagram [12].

Data extraction

At least two reviewers will independently extract data from selected studies using structured and standardised data extraction forms used in our previous qualitative and quantitative systematic reviews. In instances where multiple publications relate to the same study, these will be reported together. The following domains will be retrieved: study characteristics (authors, publication year, country, aim, study time period, study design, location, type of setting, data collection period, data

collection method, sampling strategy, analysis, participant characteristics (participant age, sample size, vaccination status of participants, socioeconomic indicators, race/ethnicity, gender, and religion) and study results (uptake of vaccine, psychological outcomes, healthcare service use, incidence of vaccine preventable disease, views and behaviours related to self-consent procedures, authors' reported conflicts of interest and study funding sources). We will also record data relating to the possible harms resulting from self-consent procedures (e.g. conflict with parents, healthcare professional anxiety). Where possible, authors will be contacted for missing or incomplete data. Disagreements will be resolved through discussion.

Risk of bias and quality assessment

For eligible primary studies, quality assessment will be undertaken to illustrate potential sources of bias. As we anticipate the majority of eligible studies will be observational, studies will not automatically be excluded on the basis of 'low' quality assessment if they are considered to contribute relevant information. We propose using: the Cochrane Collaboration's handbook for the assessment of risks of bias for systematic review of randomised controlled studies and quasi-randomised intervention studies [13] Risk Of Bias in Non Randomised Studies of Interventions (ROBINS-I) [14] ;, the NIH Quality Assessment Tool for Observational Cohort and Cross-Sectional Studies [15], and; the Critical Appraisal Skills Programme criteria adapted for qualitative studies for evaluating qualitative research [16]. Quality assessment of primary studies will be undertaken independently by two reviewers and recorded in an excel spreadsheet. An overall assessment of 'high', 'medium', 'low', or 'unclear' will be assigned and reported.

Data synthesis: Quantitative studies

We anticipate that the primary quantitative studies will be reported narratively as preliminary searches specifically related to HPV vaccination programmes indicated a lack of published studies and the likelihood of heterogeneity in relation to study design and reported outcomes. However, if

sufficiently similar studies are captured we will consider combining individual study results through meta-analyses. To assess the heterogeneity between studies, we will use the Q-statistic and the I^2-statistics [17]. Evidence of heterogeneity will be classified as weak, moderate and strong for corresponding I^2 of 25%, 50% and 75% respectively. If heterogeneity between studies is classified as weak, analyses will comprise adjusted odds ratios (aORs) where available, with unadjusted odds ratios used if not reported. Analyses will be undertaken using the meta-analysis function [18] available in Stata 15. We do not anticipate sufficient data being available to undertake sub-group analyses. However, if sufficient data were reported we propose two sub-group analyses to compare impact of self-consent procedures by: (i) setting (healthcare vs. school) and (ii) age of participants (less than 14 years old vs. 14 years and greater).

Data synthesis: Qualitative studies

The socio-ecological model [19] considers that behaviour is shaped by a complex interaction between factors operating at public policy, community, organisational, interpersonal and intrapersonal levels. In a previous qualitative synthesis, we have shown that young women's access to the HPV vaccine is shaped by decisions at different levels of the socio-ecological model [5]. During the analysis, we will use the socio-ecological model to provide a framework for understanding how barriers and facilitators operating at different levels of the model can provide access to, or prevent, young people self-consenting in the context of vaccination programmes.

To analyse the qualitative data, the methodology for thematic synthesis reported by Thomas and Harden [20], assisted by the Framework method of qualitative data management [21], will be used. These methods are suited to studies with *a priori* aims and objectives. The overall purpose of the synthesis will be to 'pool' the results from individual primary studies by initially separating the findings, coding and interpreting the text, and then combining them through the identification of key themes across the studies as well as similarities and differences within those themes [22]. Thematic

For peer review only

synthesis will be led by one reviewer reporting to the wider team about interpretation of the data as analysis progresses.

Familiarisation with the dataset will begin with reading the full papers. Pertinent sections of the text reported in each primary study will represent the basic units for analysis. Primary charts of the text will be constructed around key issues using the Framework Matrix within QSR NVivo10 software. For example, initial charts are likely to focus on 'barriers' and 'facilitators' to adolescent self-consent. The primary charts will be retained and revisited as required. Streamlined versions will be produced as the process of coding, summarising and synthesising the data progresses. In subsequent charts, key terms and phrases will be retained while repetition within studies and extraneous text are removed. During this process, overarching themes will be identified, and differences or similarities explored within these emerging themes.

Data synthesis: Interrogation

The final stage of the analysis will aim, firstly, to test whether the recommendations developed from the qualitative studies have been addressed in evaluative studies retrieved for the review and, secondly, to examine whether interventions that match the recommendations result in higher uptake in vaccination [9].

Patient and public involvement

The Bristol Young People's Advisory Group (YPAG) comprises young people aged ten to 17 years who are interested in healthcare and research. They meet regularly to help researchers with their projects and have been consulted about the design of the wider study and participant materials. They will also be invited to an event at the end of the study to consider findings and recommendations with the young people, parents, immunisation nurses and school staff involved in the study.

For peer review only

ETHICS AND DISSEMINTATION

We will not seek ethical approval for this study because the secondary data to be collected cannot be linked to individuals. As far as we are aware, this will be the first systematic review to collate evidence in relation to adolescent self-consent procedures for vaccination programmes. The review comprises part of a larger study. The findings of this review will inform the larger study evaluating the practicality, acceptability and impact of new self-consent procedures for the schools-based HPV vaccination programme in the UK. Findings will also be used to make recommendations to improve self-consent procedures for young people in vaccination programmes. We anticipate the results of this study this may be of interest to national and international stakeholders interested in improving uptake in adolescent vaccination programmes.

FULL REFERENCES

1. World Health Organisation, Summary of WHO Position Papers - Recommendations for Routine Immunization, 2017. Available from - http://www.who.int/immunization/policy/Immunization_routine_table1.pdf?ua=1.

2. Public Health England, Green Book Chapter 11: The UK Immunisation Schedule, 2016. Available from - https://www.gov.uk/government/uploads/system/uploads/attachment_data/file/554298/Green_Book_Chapter_11.pdf.

3. Convention on the Rights of the Child. United Nations, Treaty Ser, 1989. **1577**.

4. World Health Organisation, Considerations regarding consent in vaccinating children and adolescents between 6 and 17 years old, 2014. Available from - http://www.who.int/immunization/programmes_systems/policies_strategies/consent_note_en.pdf.

5. Batista Ferrer, H., et al., Barriers and facilitators to HPV vaccination of young women in high-income countries: a qualitative systematic review and evidence synthesis. BMC Public Health, 2014. **14**(1): p. 700.

6. Batista Ferrer, H., Factors Influencing the Uptake of the Human Papillomavirus (HPV) Vaccination Programme. PhD Thesis. University of Bristol, November 2014.

For peer review only

7. Batista Ferrer, H., et al., Barriers and facilitators to uptake of the school-based HPV vaccination programme in an ethnically diverse group of young women. J Public Health (Oxf), 2016. **38**(3): p. 569-577.

8. Audrey, S., et al., Impact and acceptability of self-consent procedures for the school-based human papillomavirus vaccine: a mixed-methods study protocol. BMJ Open, 2018. **8**(3).

9. Harden, A. and J. Thomas, Mixed methods and systematic reviews: examples and emerging issues. Mixed Methods Handbook, ed. A. Tashakkori and C. Teddlie. 2010, New York: Sage Publications.

10. Moher, D., et al., Preferred reporting items for systematic review and meta-analysis protocols (PRISMA-P) 2015 statement. Systematic Reviews, 2015. **4**(1): p. 1.

11. Young people's health--a challenge for society. Report of a WHO Study Group on young people and "Health for All by the Year 2000". World Health Organ Tech Rep Ser, 1986. **731**: p. 1-117.

12. Moher, D., et al., Preferred reporting items for systematic reviews and meta-analyses: The PRISMA statement. International Journal of Surgery, 2010. **8**(5): p. 336-341.

13. Higgins J, Green, S., Cochrane Handbook for Systematic Reviews of Interventions, 2011. Available from - http://handbook.cochrane.org.

14. Sterne, J.A., et al., ROBINS-I: a tool for assessing risk of bias in non-randomised studies of interventions. BMJ, 2016. **355**.

15. National Institute for Health Quality Assessment Tool for Observational Cohort and Cross-Sectional Studies, 2017. Available from - https://www.nhlbi.nih.gov/health-topics/study-quality-assessment-tools.

16. Public Health Resource Unit, Critical Appraisal Skills Programme (CASP): appraisal tools, 1998. Available from - http://www.york.ac.uk/inst/crd/index_guidance.htm.

17. Higgins, J. and S.G. Thompson, Quantifying heterogeneity in a meta-analysis. Statistics in medicine, 2002. **21**(11): p. 1539-1558.

18. Sterne, J., M.J. Bradburn, and M. Egger, Meta–Analysis in Stata™, in Systematic Reviews in Health Care. 2008, BMJ Publishing Group. p. 347-369.

19. McLeroy, K.R., et al., An ecological perspective on health promotion programs. Health education quarterly, 1988. **15**(4): p. 351-377.

20. Thomas, J. and A. Harden, Methods for the thematic synthesis of qualitative research in systematic reviews. BMC Medical Research Methodology, 2008. **8**(1): p. 45.

21. Ritchie J, Lewis .J., ed. Qualitative research practice: a guide for social science students and researchers. 2003, Sage: London.

22. Estabrooks, C.A., P.A. Field, and J.M. Morse, Aggregating qualitative findings: an approach to theory development. Qualitative Health Research, 1994. **4**(4): p. 503-511.

For peer review only

LIST OF ABBREVIATIONS

HPV: Human Papillomavirus

WHO: World Health Organisation

UK: United Kingdom

USA: United States of America

PRISMA: Preferred Reporting Items for Systematic Reviews and Meta-Analyses Protocol

PROSPERO: Prospective Register of Systematic Reviews

MeSH: Medical Subject Headings

STROBE: Strengthening the Reporting of Observational Studies in Epidemiology

aOR: Adjusted Odds Ratio

OR: Odds Ratio

YPAG: Young Person's Advisory Group

AUTHORS' CONTRIBUTIONS

All authors were involved in the conception and design of the research. SA is Principal Investigator; HB-F is study manager and lead researcher; JM and MH advise on systematic review methodology. HB-F wrote the first draft and all authors contributed to the final version of the manuscript.

FUNDING STATEMENT

This work is supported by the National Institute for Health Research for Patient Benefit (NIHR RfPB) programme (project number PB-PG-0416-20013). The work is also undertaken with the support of the NIHR Health Protection Research Unit in Evaluation of Interventions. The work was also undertaken with the support of The Centre for the Development and Evaluation of Complex Interventions for Public Health Improvement (DECIPHer), a UKCRC Public Health Research Centre of Excellence. Joint funding (MR/KO232331/1) from the British Heart Foundation, Cancer Research UK,

For peer review only

Economic and Social Research Council, Medical Research Council, the Welsh Government and the Wellcome Trust, under the auspices of the UK Clinical Research Collaboration, is gratefully acknowledged. The views and opinions expressed therein are those of the authors and do not necessarily reflect those of the NIHR RfPB Programme, the Department of Health, or Public Health England.

COMPETING INTERESTS STATEMENT

The authors declare there are no competing interests.

For peer review only

Table 1. Embase search strategy

1. child/
2. adolescent/
3. ("Young people#" OR "young person#" OR "young offender#" OR adolescent# OR adolescence OR youth# OR minor# OR teen OR teens OR teenage OR teenaged OR teenager# OR juvenile# OR pupil# OR boy# OR girl# OR underage# OR daughter# or son# (school AND dropout#) OR (school AND "drop out#") OR "school aged").mp.
4. active immunization/
5. immunization/
6. immunization programs/
7. mass immunization/
8. revaccination/
9. vaccination/
10. diphtheria vaccine/
11. diphtheria tetanus vaccine/
12. diphtheria pertussis tetanus Haemophilus influenzae type b vaccine/
13. hepatitis B vaccine/
14. meningococcus vaccine/
15. rubella vaccine/
16. wart virus vaccine/
17. Papillomavirus Vaccines/
18. (cervical cancer or diptheria or diphtheria or diphteria or DtaP or DTP or Hep B or hepatitis or HPV or measles or MenC or MenACWY or meningitis or Meningococcal or Neisseria meningitidis or papillomavirus or pertus* or rubella or rubeola or td?ipv or tetanus or wart virus or whoop*).tw.
19. (policy OR program*)

For peer review only

For peer review only - http://bmjopen.bmj.com/site/about/guidelines.xhtml

20. (immuniz* OR immunis* OR immunother* OR inoculat* OR innoculat* OR prophyla* OR revaccinat* OR vaccin*).mp.

21. Decision making/

22. Informed consent/

23. Parental consent/

24. Treatment refusal/

25. (assent* OR competen* OR decision-making OR decision making OR Gillick OR Fraser OR inform* consent OR mental capacity OR minor consent OR parent* consent OR permission* OR presume* consent OR treatment refusal OR self consent OR self-consent OR opt-out OR opt-in).mp.

26. 1 or 2 or 3

27. 4 or 5 or 6 or 7 or 8 or 9 or 10 or 11 or 12 or 13 or 14 or 15 or 16 or 17

28. 18 and 20

29. 19 and 20

30. 27 or 28 or 29

31. 21 or 22 or 23 or 24 or 25

32. 26 and 30 or 31

For peer review only

PRISMA-P 2015 Checklist

This checklist has been adapted for use with systematic review protocol submissions to BioMed Central journals from Table 3 in Moher D et al: Preferred reporting items for systematic review and meta-analysis protocols (PRISMA-P) 2015 statement. *Systematic Reviews* 2015 4:1

An Editorial from the Editors-in-Chief of *Systematic Reviews* details why this checklist was adapted - Moher D, Stewart L & Shekelle P: Implementing PRISMA-P: recommendations for prospective authors. *Systematic Reviews* 2016 5:15

Section/topic	#	Checklist item	Information reported		Line number(s)
			Yes	No	
**ADMINISTRATIVE INFORMATION**					
**Title**					
Identification	1a	Identify the report as a protocol of a systematic review	X	☐	1
Update	1b	If the protocol is for an update of a previous systematic review, identify as such	☐	X	
**Registration**	2	If registered, provide the name of the registry (e.g., PROSPERO) and registration number in the Abstract	X	☐	48, 120-21
**Authors**					
Contact	3a	Provide name, institutional affiliation, and e-mail address of all protocol authors; provide physical mailing address of corresponding author	X	☐	3-18
Contributions	3b	Describe contributions of protocol authors and identify the guarantor of the review	X	☐	362-365
**Amendments**	4	If the protocol represents an amendment of a previously completed or published protocol, identify as such and list changes; otherwise, state plan for documenting important protocol amendments	☐	X	
**Support**					
Sources	5a	Indicate sources of financial or other support for the review	X	☐	367-377
Sponsor	5b	Provide name for the review funder and/or sponsor	X	☐	367-377
Role of sponsor/funder	5c	Describe roles of funder(s), sponsor(s), and/or institution(s), if any, in developing the protocol	X	☐	367-377
**INTRODUCTION**					
**Rationale**	6	Describe the rationale for the review in the context of what is already known	X	☐	59-107

BioMed Central The Open Access Publisher

For peer review only

Section/topic	#	Checklist item	Information reported		Line number(s)
			Yes	No	
**Objectives**	7	Provide an explicit statement of the question(s) the review will address with reference to participants, interventions, comparators, and outcomes (PICO)	X	☐	32-34, 104-107
**METHODS**					
**Eligibility criteria**	8	Specify the study characteristics (e.g., PICO, study design, setting, time frame) and report characteristics (e.g., years considered, language, publication status) to be used as criteria for eligibility for the review	X	☐	147-166
**Information sources**	9	Describe all intended information sources (e.g., electronic databases, contact with study authors, trial registers, or other grey literature sources) with planned dates of coverage	X	☐	147-166
**Search strategy**	10	Present draft of search strategy to be used for at least one electronic database, including planned limits, such that it could be repeated	X	☐	382
STUDY RECORDS					
Data management	11a	Describe the mechanism(s) that will be used to manage records and data throughout the review	X	☐	145, 175-176
Selection process	11b	State the process that will be used for selecting studies (e.g., two independent reviewers) through each phase of the review (i.e., screening, eligibility, and inclusion in meta-analysis)	X	☐	168-177
Data collection process	11c	Describe planned method of extracting data from reports (e.g., piloting forms, done independently, in duplicate), any processes for obtaining and confirming data from investigators	X	☐	179-192
**Data items**	12	List and define all variables for which data will be sought (e.g., PICO items, funding sources), any pre-planned data assumptions and simplifications	X	☐	183-192
**Outcomes and prioritization**	13	List and define all outcomes for which data will be sought, including prioritization of main and additional outcomes, with rationale	X	☐	183-192
**Risk of bias in individual studies**	14	Describe anticipated methods for assessing risk of bias of individual studies, including whether this will be done at the outcome or study level, or both; state how this information will be used in data synthesis	X	☐	194-205
DATA					
**Synthesis**	15a	Describe criteria under which study data will be quantitatively synthesized	X	☐	207-220
	15b	If data are appropriate for quantitative synthesis, describe planned summary measures, methods of handling data, and methods of combining data from studies, including any planned exploration of consistency (e.g., I^2, Kendall's tau)	X	☐	207-220

BioMed Central The Open Access Publisher

3

Section/topic	#	Checklist item	Information reported		Line number(s)
			Yes	No	
	15c	Describe any proposed additional analyses (e.g., sensitivity or subgroup analyses, meta-regression)	X	☐	217-220
	15d	If quantitative synthesis is not appropriate, describe the type of summary planned	X	☐	208-210, 222-254
**Meta-bias(es)**	16	Specify any planned assessment of meta-bias(es) (e.g., publication bias across studies, selective reporting within studies)	☐	X	
**Confidence in cumulative evidence**	17	Describe how the strength of the body of evidence will be assessed (e.g., GRADE)	☐	X	

For peer review only

BioMed Central
The Open Access Publisher
